# Advancements and Applications of Artificial Intelligence and Machine Learning in Material Science and Membrane Technology: A Comprehensive Review

**DOI:** 10.3390/membranes15120353

**Published:** 2025-11-24

**Authors:** Simin Nazari, Amira Abdelrasoul

**Affiliations:** 1 Division of Biomedical Engineering, University of Saskatchewan, 57 Campus Drive, Saskatoon, SK S7N 5A9, Canada; 2 Department of Chemical and Biological Engineering, University of Saskatchewan, 57 Campus Drive, Saskatoon, SK S7N 5A9, Canada

**Keywords:** data-driven modeling, fouling prediction, membrane process optimization, material discovery, machine learning algorithms

## Abstract

Membrane technologies play a vital role in sustainable development due to their efficiency in separation, purification, and chemical processing applications. However, the discovery and optimization of new membrane materials remain largely reliant on trial-and-error experimentation, limiting the pace of innovation. Artificial intelligence (AI) and machine learning (ML) are increasingly being applied to overcome these limitations by enabling data-driven insights, predictive modeling, and rapid material design. These computational approaches have shown significant promise in accelerating membrane fabrication, improving process simulation, detecting and mitigating fouling, and enhancing membrane characterization. This review provides a comprehensive overview of the recent advancements in the integration of AI and ML within membrane and material science. Fundamental AI and ML concepts relevant to membrane science are discussed, together with their applications in membrane fabrication, performance prediction, process modeling, fouling control, and membrane design. Challenges related to data quality, model interpretability, and the integration of domain-specific knowledge are also highlighted, along with potential future research directions. Compared with conventional empirical approaches, the advantages of AI and ML in handling complex, multivariate datasets and accelerating innovation are demonstrated. Overall, this review underscores the transformative potential of AI and ML in developing next-generation membranes with improved efficiency, selectivity, and sustainability across various industrial applications. Although several reviews have explored ML applications in membrane processes, comprehensive integration across material design, fabrication, fouling control, optimization, and process modeling remains limited.

## 1. Introduction

Membrane technology constitutes a cornerstone of modern separation and purification systems, forming an indispensable part of water and wastewater treatment, biomedical applications such as hemodialysis, and various industrial and energy-related processes [1,2,3,4]. Membranes act as selective barriers that enable efficient separation of particles, molecules, or ions based on size, charge, or affinity, and are fabricated from a broad spectrum of materials, including polymeric membranes (e.g., polyethersulfone, polysulfone, polyvinylidene fluoride, and polyamide) as well as inorganic and hybrid composites [5,6,7,8,9,10,11,12,13]. Although membranes are essential in these applications, their performance and long-term stability are strongly governed by the physicochemical characteristics of the base materials and surface structures [14,15,16,17]. Designing membranes with optimal permeability, selectivity, mechanical strength, and antifouling properties requires careful control of their material composition, porosity, and hydrophilicity/hydrophobicity balance.

Material science and membrane technology are two closely intertwined fields that have brought significant transformations in diverse industrial processes and applications. Material science focuses on the study of the structure, properties, and synthesis of materials to develop new materials with enhanced functionalities and performance [18,19,20,21]. Membrane technology, on the other hand, involves the use of selectively permeable membranes to separate and purify substances, control the flow of fluids, and facilitate various chemical processes [22,23,24,25,26]. In membrane technology, materials play a crucial role as the building blocks of membranes [23,27,28]. The selection of appropriate materials is vital to achieving desired properties and durability.

However, material science and membrane technology face several challenges that can benefit from the application of AI and ML. Some of these challenges include material discovery, data-driven analysis, structure-property relationships, multivariate optimization, fouling prediction and its mitigation, process control, and optimization [29,30,31,32,33]. The integration of AI with material science and membrane technology has yielded substantial effects and results. AI algorithms and techniques have been applied to accelerate materials discovery and design, revolutionizing the field of material science [34,35,36,37,38]. Through ML and data analytics, AI can efficiently analyze large datasets, identify patterns, and predict material properties, leading to the discovery of novel materials with tailored properties for specific applications [34,39,40,41,42,43,44,45]. This has significantly reduced the time and cost required for materials development, enabling the design of advanced membranes with enhanced performance characteristics [39,44,46,47,48].

Moreover, AI and ML can also be used to develop predictive models that analyze various factors such as operating conditions, feed composition, and membrane properties to detect and predict fouling events [49,50]. This can aid in implementing proactive fouling mitigation strategies and improving membrane lifespan. Additionally, AI-powered algorithms can optimize membrane fabrication processes, ensuring precise control over membrane structure, porosity, and surface properties. This integration has facilitated the development of high-performance membranes with improved selectivity, permeability, and stability. Furthermore, AI-based control and monitoring systems have enhanced the efficiency and reliability of membrane-based processes by enabling real-time optimization, fault detection, and predictive maintenance. Overall, the synergy between AI, material science, and membrane technology has unlocked new opportunities for innovation, enabling the development of advanced materials and membranes that contribute to improved industrial processes and applications [34,51,52,53,54].

In summary, the incorporation of ML and AI into the domains of membrane technology and material design has brought about a profound transformation in the field of materials science. This integration allows researchers to surmount the constraints inherent in conventional research methods and propel the advancement of highly efficient membrane technologies possessing the desired properties [55,56,57,58,59,60]. This review builds upon our prior research [61,62,63,64,65,66,67,68], providing further insights into the transformative impact of AI and ML on material science and especially membrane technology. By continuously pushing the boundaries of what is possible, AI and ML continue to drive the field towards greater achievements, ultimately shaping the future of materials science and revolutionizing various industries.

Despite the growing number of research addressing the role of AI and ML in membrane science, most existing studies have focused on specific subtopics, such as fouling prediction, process control, or other particular membrane processes [69,70,71,72]. However, there remains a lack of a comprehensive and integrative review that connects the application of AI and ML across the full membrane lifecycle, from material discovery and fabrication to process simulation, fouling control, and optimization. Moreover, prior reviews often emphasize algorithmic performance rather than explaining how AI/ML can accelerate material design, interpret structure–property relationships, and enhance sustainable membrane innovation. Another key limitation in previous literature is the fragmented perspective between material science and membrane engineering and their applications. While materials informatics reviews [73,74] have discussed ML for molecular or polymer design, they rarely link these methods to membrane performance metrics, such as selectivity, permeability, roughness, and hemocompatibility. Therefore, this review aims to bridge these interdisciplinary domains by systematically examining recent advancements in the integration of AI and ML within both material science and membrane technology. The synergistic role of AI and ML in transforming membrane research is emphasized through their ability to accelerate fabrication, enhance process modeling, mitigate fouling, and improve membrane design and performance. As these applications are inherently interconnected, they are categorized in this review into six overlapping domains: membrane technology, fabrication, process modeling, fouling control, design and characterization, and future challenges. By integrating insights from material informatics, process engineering, and membrane science, a unified framework is presented to demonstrate how AI and ML drive the development of next-generation membranes.

The objectives of this review are to provide a comprehensive overview of recent advancements in AI and ML applications within material science and membrane technology, and to examine how these approaches accelerate progress across the full membrane lifecycle—including fabrication, process modeling, fouling prediction, and membrane characterization. In doing so, this review analyzes and compares various AI and ML algorithms to assess their effectiveness in addressing key challenges in membrane research. It also highlights current limitations and data-related issues while emphasizing the need for hybrid and interpretable AI models to improve reliability and scientific insight. Finally, the review proposes future research directions and presents a unified framework that integrates AI and ML tools with experimental and theoretical methodologies to drive innovation in next-generation membrane systems.

## 2. Literature Search Methodology

To ensure comprehensive coverage of relevant studies, a structured literature search was conducted across Scopus, Web of Science, ScienceDirect, PubMed, and Google Scholar databases. The search included publications from 2015 to 2025 using combinations of keywords such as “artificial intelligence”, “machine learning”, “deep learning”, “membrane”, “material science”, “fabrication”, “fouling”, “optimization”, and “simulation”. Only peer-reviewed journal articles written in English were considered. Review papers, conference abstracts, and studies not directly related to AI/ML applications in material science or membrane technology were excluded. The selected literature was further categorized based on its focus area—material design, membrane fabrication, process modeling, fouling detection, and performance optimization—to support the integrative structure of this review.

## 3. AI and ML Paradigms and Their Roles in Membrane Technology

AI and ML have emerged as powerful computational tools capable of discovering complex, nonlinear relationships within high-dimensional datasets. In membrane and material science, these methods enable researchers to predict material properties, optimize fabrication parameters, and design membranes with improved permeability, selectivity, and antifouling performance. Instead of relying solely on trial-and-error experiments, ML-driven models can analyze large datasets of polymer composition, surface roughness, contact angle, and protein interactions to identify patterns that guide the rational design of advanced membranes. These approaches not only reduce development time and cost but also support the design of next-generation membranes with tailored surface properties and enhanced selectivity.

ML encompasses several subfields, each offering distinct advantages for membrane-related applications [34,35,36,37,38,75,76]. Supervised learning is widely used to predict quantitative membrane properties such as flux [77], salinity rejection rate [78,79], and surface-related parameters based on experimental inputs [73,80,81,82]. Regression and classification models have been effectively employed for these purposes. Unsupervised learning helps uncover hidden structures in complex datasets—for example, clustering membranes based on morphology, roughness, or pore size distribution, or identifying relationships among fabrication parameters that influence fouling behavior [83]. Semi-supervised learning becomes valuable when limited labeled data are available, allowing researchers to combine experimental measurements with simulation or computational data to enhance predictive accuracy and generalization.

Reinforcement learning and deep learning represent more advanced ML paradigms increasingly applied to membrane research. Reinforcement learning supports process control and dynamic optimization by enabling models to autonomously adjust operational parameters (e.g., pressure, feed flow rate, backwashing frequency) to minimize fouling and energy consumption [84,85]. Deep learning, particularly convolutional neural networks (CNNs) and recurrent neural networks (RNNs), has been employed for image-based membrane characterization and fouling detection using SEM, AFM, or synchrotron datasets [70,86,87]. Furthermore, backpropagation-based neural networks (BPNNs) have been applied to predict fouling behavior from spectral data, demonstrating that deep architectures can also process non-image features [88]. Each of these approaches contributes uniquely to advancing membrane design, operation, and performance prediction (Table 1). Among these, Random Forest, XGBoost, CNN, and RNN models are the most widely applied ML frameworks in membrane science, demonstrating strong predictive power across regression, classification, and image-based prediction tasks. Meanwhile, BPNNs, unsupervised clustering models (PCA–GMM), and reinforcement learning algorithms are emerging as promising tools for complex feature extraction, fouling pattern recognition, and process control (Table 1).

**Table 1:** Summary of representative applications of various ML models in membrane and material science.

ML Model	Learning Type	Membrane Application	Input Features	Output	Performance Metrics	Ref.
Random Forest	Supervised (Regression)	Predicting water flux in osmotic membrane bioreactor (OMBR)	Phosphate, MLSS, TOC, NH_4_, Influent phosphate	Water flux	R^2^ = 0.987, RMSE = 0.044	[77]
XGBoost	Supervised (Regression)	Prediction of organic contaminant rejection rate in nanofiltration (NF) and reverse osmosis (RO) membranes	MWCO, molecular weight (MW), McGowan volume (V), contact angle (CA), total charge (TC), pressure (P), initial concentration (Cin), pH, hydrogen bond basicity (B), and time (T)	Rejection rate (%)	R^2^_adj = 99.5%, R^2^_ext = 87.3%, RMSE = 1.674, MAE = 1.065	[78]
CatBoost	Supervised (Regression)	Prediction of water permeability (A) in RO membranes	Membrane composition (structure, chemistry, modification), concentration polarization modulus (CP), and pressure difference (ΔP)	Water Permeability (A, L·m^−2^·h^−1^·bar^−1^)	R^2^_adj = 0.925, MAE = 0.246	[79]
Extremely Randomized Trees (ET)	Supervised (Regression)	Prediction of solute permeability (B) in RO membranes	Membrane composition, R_real, and CP	Solute permeability (B, L·m^−2^·h^−1^)	R^2^_adj = 0.986, MAE = 0.069	[79]
Logistic Regression	Supervised (Classification)	Predictive classification of pore size in regenerated cellulose (RC) membranes using AFM data	AFM surface parameters (pore radius, scan area, skewness, kurtosis, Fourier-fit data, imaging mode: tapping/fluid)	Pore size class (50 kDa, 100 kDa, 1000 kDa)	AUC = 0.83 (tapping), 0.76 (fluid); Accuracy ≈ 76–77%	[80]
Random Forest	Supervised (Regression)	Quantitative prediction of protein adsorption (BSA, lysozyme) on polymer brush surfaces	Polymer brush thickness (nm), water contact angle (°), ζ potential (mV), and molecular descriptors (hydrophobicity, polarity, and surface charge parameters)	Adsorption amount (ng/cm^2^) for BSA and lysozyme	R^2^ = 0.94 (BSA); lower accuracy for lysozyme	[81]
Random Forest	Supervised (Regression)	Prediction of Affinity Energy between human serum proteins and hemodialysis membrane materials	12 molecular descriptors (e.g., number of atoms, carbon, nitrogen, oxygen, sulfur, MW, aromatic rings, charged groups, H-bond donors/acceptors, protein type indicators)	Affinity Energy (kcal/mol)	R^2^ = 0.8987, MSE = 0.36, MAE = 0.45	[82]
Gaussian Mixture Model (GMM) + Principal Component Analysis (PCA)	Unsupervised (Clustering)	Morphology clustering of polyamide membranes	TEM morphology shape fingerprints	Morphology clusters	BIC used for cluster validation	[83]
Proximal Policy Optimization (PPO) − Deep Reinforcement Learning (DRL) with LSTM environment	Reinforcement Learning (Process control and dynamic optimization)	Ultrafiltration system operation optimization	Feed pressure, cleaning time, cleaning concentration, and system states (flux, turbidity, conductivity, temperature)	Optimized operating policy to maximize water flux and reduce energy consumption	Reduction in specific energy consumption (SEC) by 20.9%, with average flux increased (39.5 → 43.7 L·m^−2^·h^−1^)	[84]
CNN	Deep Learning (Supervised Regression)	Prediction of membrane fouling	Hyperspectral image data	Fouling indices	R^2^ = 0.71; MSE = 435.21	[87]
BPNN	Supervised (Regression)	Prediction of Unified Membrane Fouling Index (UMFI)	UV–Vis and EEM fluorescence, and synchrotron fluorescence spectra	UMFI (fouling potential)	R^2^ = 0.965, RMSE = 0.002	[88]

**Figure 1:**
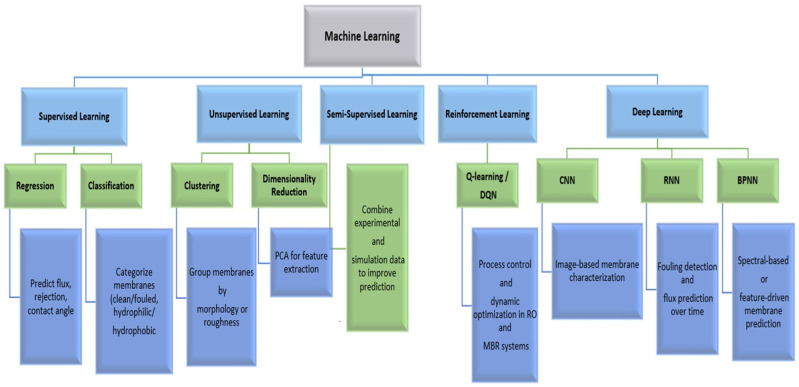
Various ML models and their applications in membrane technology. Adapted and modified from Nassif et al. [96] (2019), with permission under the Creative Commons Attribution 4.0 License (CC BY 4.0).

To illustrate the role of these ML approaches, Figure 1 presents an integrative framework linking different ML methods with their corresponding membrane applications. This conceptual diagram demonstrates how supervised, unsupervised, and deep learning techniques are utilized across various stages of the membrane lifecycle—from material selection and fabrication to fouling monitoring and process optimization. Such frameworks highlight the versatility of AI/ML methods in addressing diverse challenges in both material and membrane science, setting the stage for the comparative analyses in the following sections.

As summarized in Table 1, model performance and validation strategies vary depending on the complexity of the data and the prediction target. Ensemble models such as Random Forest and XGBoost generally yield the highest accuracy (R^2^ > 0.85) for regression-based membrane property predictions, while CNNs and RNNs demonstrate strong performance in image-based and time-dependent analyses. Most studies evaluated model reliability using separate test sets or k-fold cross-validation and reported metrics such as R^2^, RMSE, and MAE. Reinforcement learning algorithms have shown potential for dynamic process control, though their validation remains qualitative in most cases [89,90,91,92,93,94,95,96]. Overall, these comparative results emphasize that the choice of ML model depends on the type of input data, problem complexity, and the level of interpretability required.

## 4. AI and ML in the Membrane Fabrication

**Figure 2:**
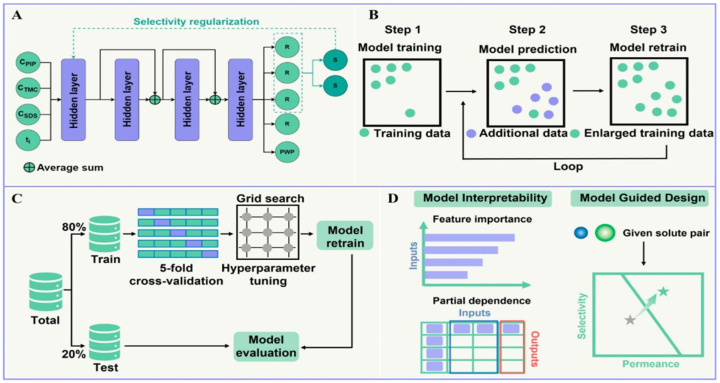
Schematic diagram of (**A**) Network structure of the multitask MLP, (**B**) Four-round online learning process, (**C**) Model training and evaluation, and (**D**) Model’s interpretation in membrane design [110]. Reproduced with permission from Deng H. et al., Environ. Sci. Technol. (2023), 57, 17841-17850, Copyright © 2023 American Chemical Society. License number: 6116070593357.

The development of membrane materials for various separation processes has traditionally relied on a time-consuming trial-and-error approach, leading to slow progress [97,98,99]. Polymer membranes, although sustainable and easy to fabricate, face trade-offs in permeability and selectivity and have limited resistance to high temperature and pressure [100,101]. The complexity of the polymer space and the variability introduced by mixed-matrix membranes further complicate the material selection and fabrication process [102,103,104]. However, the emergence of AI and ML offers new opportunities for faster and more efficient membrane design. ML models can analyze large datasets generated from material synthesis and data collection techniques, enabling the discovery and rational design of high-performance materials [75,105,106]. ML has already shown success in predicting polymer properties and understanding the relationships between material properties and structures. J.W. Barnett et al. [107] proposed a novel approach to polymer membrane design using ML algorithms. The authors trained an ML algorithm using a topological, path-based hash of the polymer repeating unit. Instead of conducting exhaustive experimental investigations, they utilized a limited set of experimental gas permeability data for six different gases in approximately 700 polymeric constructs. By leveraging this dataset, they were able to predict the gas separation behavior of over 11,000 homo-polymers that had not been previously tested for these properties. The accuracy of the ML algorithm was validated by synthesizing two of the most promising polymer membranes predicted by the model, which exhibited superior CO_2_/CH_4_ separation performance beyond the established upper bound. This approach demonstrates the potential of ML in exploring the vast design space of polymer membranes and predicting optimal materials for specific applications, even with limited experimental data. M. Tan et al. [108] used hybrid models based on a back propagation neural network (BPNN) and genetic algorithm (GA) to optimize the fabrication of polyetherimide (PEI) ultrafiltration (UF) membranes using the dry/wet phase inversion method. The BPNN was employed to establish the relationships between preparation conditions and membrane performances, while GA was used to determine the initial connection weights and biases of the BPNN to prevent convergence at suboptimal solutions. The hybrid models showed excellent agreement with testing data, indicating their accuracy. By using the hybrid models, the effects of preparation conditions on membrane performances were successfully predicted, identifying PEI/NN-dimethylacetamide (DMAc)/1,4-butyrolactone (GBL) as the optimal membrane casting system. Additionally, the hybrid models accurately forecasted the optimal preparation conditions and enabled the fabrication of membranes with desired performances, such as higher pure water flux (PWF) and bovine serum albumin (BSA) rejection ratio (RR) of 80–90%, with a small deviation between predicted and experimental values. In another study by B. Li et al. [109], the development of high-performance membranes for resolving water pollution using AI is addressed. The focus is on the application of electroless nickel plating (ENP) as a membrane fabrication technique. The complexity and multiple factors involved in ENP hinder its optimization. To overcome this, the study proposes a method that integrates the response surface method (RSM) with AI to optimize ENP conditions for polyvinylidene fluoride-nickel (PVDF-Ni) membrane fabrication. RSM simulation based on experimental data identifies the optimal conditions, and the combined RSM-artificial neural network (ANN) method is used to predict membrane properties. H. Deng et al. [110] utilized AI and ML techniques to design polymeric membranes with high solute-solute selectivity and permeance. Their novel ML model, based on an ANN with skip connections and selectivity regularization, was trained using limited lab-collected data. The model outperformed other ML approaches, exceeding the upper bound of selectivity and permeance for mono/divalent ions. Mechanistic insights were obtained through feature analysis. The model generalization capability, based on MLP, effectively connected fabrication conditions and membrane performance, offering a scalable and efficient approach for membrane design. The model-guided membranes demonstrated exceptional performance, surpassing current state-of-the-art membranes (Figure 2).

**Figure 3:**
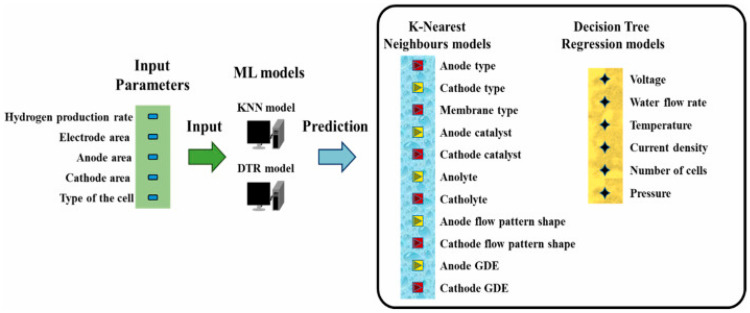
Chart illustrating the ML models, showcasing five shared input parameters and unique output parameters for each model [111]. Reproduced with permission from Yang R. et al., Energy (2023) 264, 126135, Copyright © 2022 Elsevier Ltd. License number: 6116070088788.

R. Yang et al. [111] investigated the design and optimization of a proton exchange membrane (PEM) electrolyzer for green hydrogen production using ML techniques. The proposed ML model employed k-nearest neighbors and decision tree regression algorithms to predict the optimal design factors and flow-path shapes for the PEM electrolyzer. The model is trained and validated using 1062 design data points, achieving an absolute mean square error of 0.31 when compared to experimental results. The ML model successfully predicts 17 parameters for the electrolyzer assembly based on five input parameters, including hydrogen production rate, electrode area, anode and cathode flow areas, and cell design type (Figure 3). The study demonstrates the reliability and efficiency of the ML model in predicting the optimal design of PEM electrolyzers for commercial-scale hydrogen production rates.

**Figure 4:**
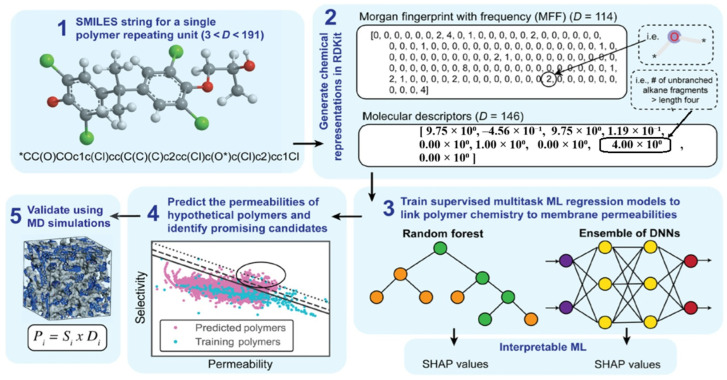
Step-by-step procedure of applying ML in the identification of novel polymer membranes with outstanding gas separation properties [112]. Reproduced with permission from Yang et al., Sci. Adv. 8, eabn9545 (2022) under the terms of the Creative Commons Attribution Noncommercial License 4.0 (CC BY-NC), published by the American Association for the Advancement of Science (AAAS).

**Figure 5:**
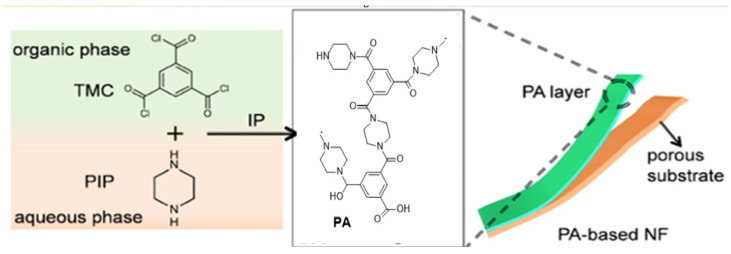
Interfacial polymerization using ML: the creation of thin-film nanocomposite (TFN) nanofiltration membranes using piperazine and trimesoyl chloride on a porous substrate [113]. Reprinted with permission from Gao, H. et al., Environ. Sci. Technol. (2022), 56, 2572−2581; Copyright © 2022, American Chemical Society. License number: 6116080725213.

J. Yang et al. [112] presented an ML approach for discovering innovative polymers with optimal performance in gas separation membranes. They trained to multitask ML models using experimental data to predict the gas permeabilities of various gases. Through interpretation of the ML models, they extracted insights into the chemical moieties that contribute to permeability and selectivity. Using these insights, they screened over 9 million hypothetical polymers and identified thousands of promising candidates that surpass current performance upper bounds (Figure 4). H. Gao et al. [113] introduced a groundbreaking method for designing polymeric membranes using ML and Bayesian optimization. The authors highlighted that the process of creating polymeric membranes is intricate and multidimensional, requiring the careful selection and optimization of membrane materials and manufacturing conditions from a seemingly endless pool of possibilities. The authors created ML models capable of accurately predicting water permeability and salt rejection based on the types of membrane monomers and their manufacturing conditions. They then utilized Bayesian optimization on these models to pinpoint the best combinations of monomers and manufacturing conditions that could potentially exceed the current limitations for water/salt selectivity and permeability. The study shows that ML and Bayesian optimization could revolutionize the design of separation membranes for the next generation. Figure 5 depicts the intricate process of forming the PA layer, which demands meticulous experimental control, including the selection and concentration of monomers, polymerization time, heat curing time, the inclusion of nanomaterials, the addition of additives, and the selection of substrate types. This figure emphasizes the necessity of ML in membrane design, as it can help navigate this complex process and identify the optimal combinations of factors for superior membrane performance.

## 5. AI and ML for Membrane Process Modeling and Simulation

One of the areas where ML and AI have shown great potential is in membrane process modeling and simulation [49,60]. Membrane processes, such as reverse osmosis, ultra-filtration, and nano-filtration, are widely used for molecular separation in various industries, including water treatment, pharmaceuticals, and food processing. The accurate modeling and simulation of these processes play a crucial role in optimizing their efficiency, designing new membranes, and improving overall performance. ML techniques such as decision trees, random forests, support vector machines, and neural networks have been successfully applied in membrane process modeling [114,115,116]. These models can effectively capture the effects of various factors, including feed composition, operating conditions, membrane properties, and fluid dynamics, on process performance. In addition to conventional pressure-driven processes, recent studies have explored the integration of ML within membrane bioreactor (MBR) systems. MBRs represent a rapidly advancing area of membrane technology that integrates biological wastewater degradation with membrane filtration, enabling the production of high-quality effluent while reducing excess sludge generation and system footprint. However, challenges such as membrane fouling and high energy demand remain critical. Recent work has demonstrated the sustainability and process optimization benefits of MBRs for wastewater treatment, reflecting the expanding role of AI-driven modeling in bioreactor membrane technologies [117,118,119]. Afterward, it is worth noting that ML algorithms can continuously learn and adapt from new data, improving the predictive capabilities of the models over time.

**Figure 6:**
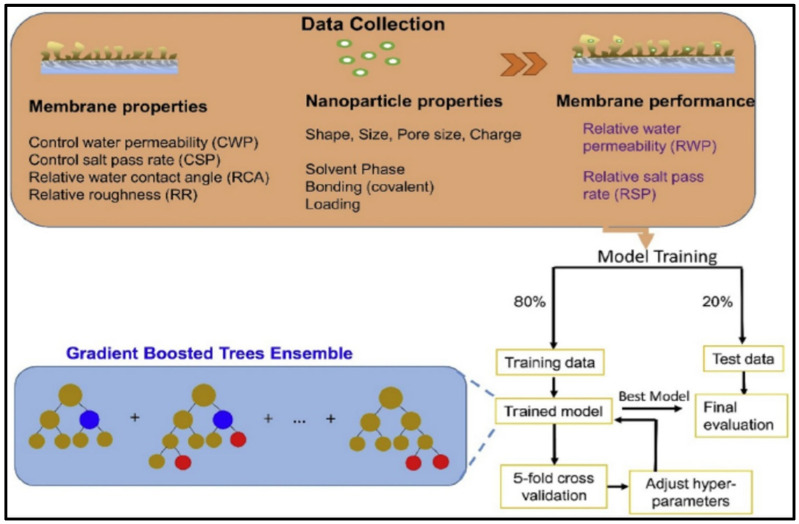
Visual representation of the input and output variable [120]. Reproduced with permission from Yeo C. et al., Journal of Membrane Science (2020), 606, 118135, Copyright © 2020 Elsevier. License number: 6060250528613.

C. S. H. Yeo et al. [120] showed the importance of ML in optimizing the water permeability and salt rejection of thin film nanocomposite (TFN) membranes used in reverse osmosis (RO) desalination. The study utilized ML techniques, specifically gradient boosting tree models, to predict the water permeability and salt pass rate of TFN membranes based on various parameters such as nanoparticle loading, size, pore size, and membrane properties (Figure 6). The results demonstrated that factors like loading, size, and hydrophilicity significantly influence membrane performance. The paper also discusses the optimization of these parameters and compares the optimized properties with aquaporin-based membranes, providing valuable insights for future membrane development. The study found that porous nanoparticles generally outperform nonporous ones in terms of membrane performance. By optimizing these factors using partial dependence plot analysis, the researchers were able to identify ways to enhance the water permeability and salt rejection of TFN membranes, providing valuable engineering guidelines for further membrane development. Given the complexity and vast range of nanoparticles and membrane fabrication parameters involved, ML proves to be a powerful tool in this context, enabling a comprehensive understanding and engineering guidelines for TFN membrane preparation and advancement.

**Figure 7:**
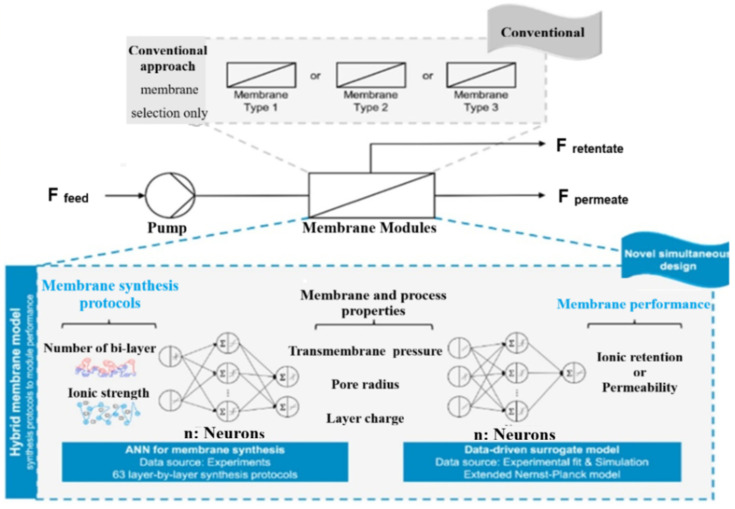
Simultaneous optimization of performance and process using ANNs [122]. Reproduced with permission from Rall D. et al., Journal of Membrane Science (2020), 600, 117860., Copyright © 2020 Elsevier. License number: 6060250983018.

D. Rall et al. [121] illustrated the importance of innovative membrane technologies in large-scale separation process plants for effective and cost-efficient water treatment. As the existing models describing mass transport through membranes are limited to the nano-scale and cannot address the complexities of large-scale processes, the authors propose the integration of artificial neural networks (ANNs) as surrogate models in deterministic global optimization. By training the ANNs on data generated from ion transport models, they achieve accurate transport models and enable multi-scale optimization for membrane processes. The developed models and optimization solver are open-source, allowing for resource-efficient and comprehensive optimization in membrane science. The methodology can be applied to various membrane science fields and provides a valuable tool for research and industrial applications. In another study, D. Rall et al. [122] proposed an optimization strategy that combines ML techniques with the design of nano-filtration membrane modules for water treatment processes. By integrating membrane synthesis into a mechanistic process model using ANNs, they achieve optimal performance and cost-effectiveness. The approach yields improved separation performance at lower costs compared to conventional strategies. The methodology offers a promising solution for designing membrane treatment plants, enabling non-intuitive solutions and more efficient piloting (Figure 7).

**Figure 8:**
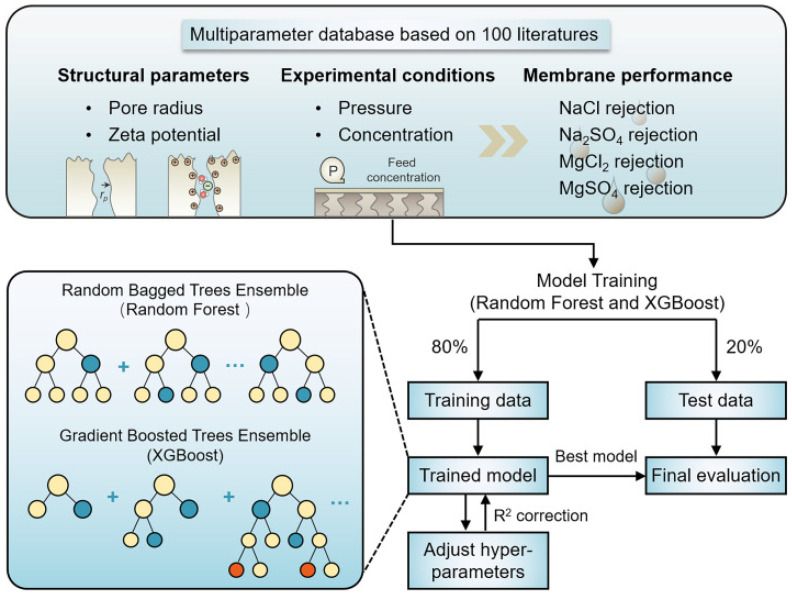
Utilizing Random Forest and XGBoost ML models to unveil the relationship between structural parameters, experimental conditions, and NF membrane performance [123]. Reproduced with permission from Ma X. et al., Desalination (2023), 548, 116293, Copyright © 2023 Elsevier. License number: 6060251471288.

X. Ma et al. [123] utilized ML to investigate the correlation between membrane structure parameters, operating conditions, and water/salt selectivity in nanofiltration (NF) membranes. The authors extracted two structural features (pore radius and zeta potential) and two operating parameters (pressure and feed concentration) and associated them with water/salt selectivity. By employing Random Forest and XGBoost models, they assessed the importance of these variables and established structure-performance relationships (Figure 8). The results revealed that membrane structure parameters played a more crucial role in water/salt selectivity than operating conditions, with different mechanisms governing symmetric and asymmetric salts.

D. Lin et al. [124] explored the application of ML models in describing the separation of liquids through a membrane system. The authors utilized computational fluid dynamics (CFD) simulations as inputs to the models, with coordinates (r and z) representing the inputs and the organic component content in the membranes feed section (C) as the single output. The dataset consists of over 8K rows, each containing two inputs and one output. Three ML models were considered: Decision Tree, Poisson regression, and K-nearest neighbors, with hyper-parameters tuned using grid search. The Decision Tree model achieved the highest accuracy, with an R^2^-score of 0.9969, while the other models also performed well. In general, the integration of CFD simulations with ML models has shown particular promise in membrane process modeling. CFD simulations provide detailed information about fluid flow, mass transfer, and other physical phenomena within the membrane system. By coupling this information with ML algorithms, a comprehensive understanding of the complex interactions between process variables and membrane molecular separation can be achieved. U. M. Ismail et al. [125] developed a data-driven framework to optimize membrane bioreactor (MBR) performance for wastewater treatment by predicting chemical oxygen demand (COD) removal efficiency. They evaluated 23 influent and operational variables from a full-scale MBR plant in Saudi Arabia and, through correlation analysis, reduced them to seven key inputs. Three algorithms—random forest regression (RFR)**,** Gaussian process regression (GPR), and stepwise linear regression (SWLR)—were compared, with RFR showing the best baseline performance (overall R^2^ ≈ 0.78). The model was further enhanced using meta-heuristic optimization techniques, including particle swarm optimization, genetic algorithm, and simulated annealing, where the GA-optimized RFR achieved the best performance, reducing prediction error and improving model reliability. The study demonstrates that integrating ML with meta-heuristic optimization can significantly enhance predictive performance and enable smarter, more sustainable operation of wastewater treatment plants.

Another important application of AI and ML in membrane technology lies in the optimization and design of feed spacers in reverse osmosis (RO) and nanofiltration (NF) systems [126,127]. Feed spacers play a vital role in maintaining the flow channel between membrane sheets [128], promoting turbulence [126], and enhancing mass transfer [127,128]. Their geometry, thickness, and filament arrangement critically influence hydrodynamics, shear stress distribution, concentration polarization, and fouling behavior, which in turn affect overall flux and energy efficiency [129]. By integrating computational fluid dynamics (CFD) data with convolutional neural networks and other deep learning architectures, researchers have developed data-driven surrogate models capable of accurately simulating flow and concentration fields at a fraction of the computational cost. M. A. Al-Obaidi et al. [126] developed a hydrodynamic and mass-transfer simulation model using gPROMS to analyze the influence of feed-spacer geometry on reverse osmosis (RO) performance during the removal of dimethylphenol from wastewater. The model simultaneously solved the Navier–Stokes, continuity, and solute-transport equations, incorporating correlations for friction factor and mass-transfer coefficient to capture the effects of spacer height, filament angle, and porosity on flow resistance and solute rejection. Model predictions were validated against experimental data, achieving an average relative error below 5%, confirming strong predictive capability. The simulations revealed that smaller spacer heights and lower filament angles enhanced mixing and concentration-polarization control, leading to higher solute rejection (up to 99.5%) and lower specific energy consumption (≈3.37 kWh m^−3^). Among the configurations tested, the UF-3 parallelogram-type spacer provided the best trade-off between flux enhancement and pressure-drop minimization. Although the approach was physics-based rather than AI-driven, its high accuracy and parameter sensitivity make it a valuable reference for future machine-learning-assisted spacer optimization studies. In another study, J. Luo et al. [130] applied a supercomputing- and machine-learning-aided optimization framework to improve the design of seawater reverse osmosis (SWRO) systems, integrating CFD with multilayer artificial neural networks (MLN) as surrogate models. The approach enabled rapid prediction of key transport parameters—pressure drop, mass-transfer coefficient, and porosity—across a wide range of feed-spacer geometries and membrane configurations. The MLN models achieved high predictive accuracy while reducing computational cost by several orders of magnitude compared with full CFD simulations. Multi-scale optimization using these models identified spacer designs that enhanced mass transfer by approximately 21% and reduced specific energy consumption by 27.5% and membrane area by 37% compared with commercial configurations. This study highlights the potential of hybrid CFD–ML frameworks to accelerate spacer geometry optimization and drive energy-efficient, high-performance RO process design.

## 6. AI and ML for Membrane Fouling Detection and Control

**Figure 9:**
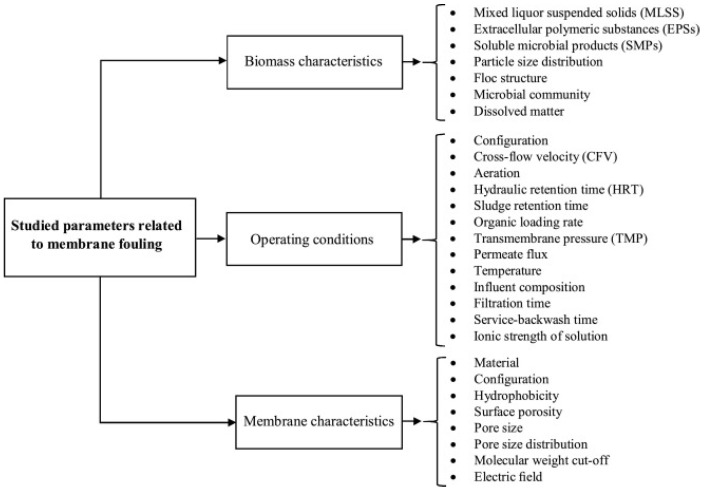
Key factors contributing to membrane fouling [49]. Reproduced with permission from Bagheri M. et al., Process Safety and Environmental Protection (2019), 123. 229–252. Copyright © 2019 Elsevier. License number: 6060270343081.

AI and ML have significantly improved the understanding of membrane fouling mechanisms by analyzing complex data patterns and identifying key factors that contribute to fouling. This knowledge has enabled the development of advanced fouling prediction models, leading to more effective fouling control strategies and the design of fouling-resistant membranes, ultimately enhancing the overall efficiency and lifespan of membrane systems. The successful application of AI and ML techniques in controlling and managing membrane fouling has been demonstrated in various studies. By integrating AI and ML techniques into advanced control systems, treatment costs can also be reduced through efficient monitoring and timely actions to address fouling issues [49,59,131]. F. Schmitt et al. [132] conducted a comprehensive review on the use of ANNs as AI techniques for predicting membrane fouling in membrane bioreactors (MBRs) used in wastewater treatment. Their findings highlighted the effectiveness of ANNs in accurately forecasting membrane fouling in filtration systems, particularly within MBR plants. The authors emphasized the importance of optimizing experimental databases and ANN architecture. They also suggested the inclusion of operational time as a parameter to expand the applicability of membrane fouling prediction using ANNs to large-scale MBRs. To effectively model and optimize membrane fouling in MBRs, it is crucial to have a comprehensive understanding of the key factors that contribute to this phenomenon. These contributing factors are typically regarded as inputs for intelligent models and optimization techniques, enabling improved control of membrane fouling. As it is shown in Figure 9, Bagheri et al. [49] have categorized the significant contributing factors to membrane fouling into three major classes: operating conditions, biomass properties, and membrane characteristics, serving as inputs for intelligent models.

**Figure 10:**
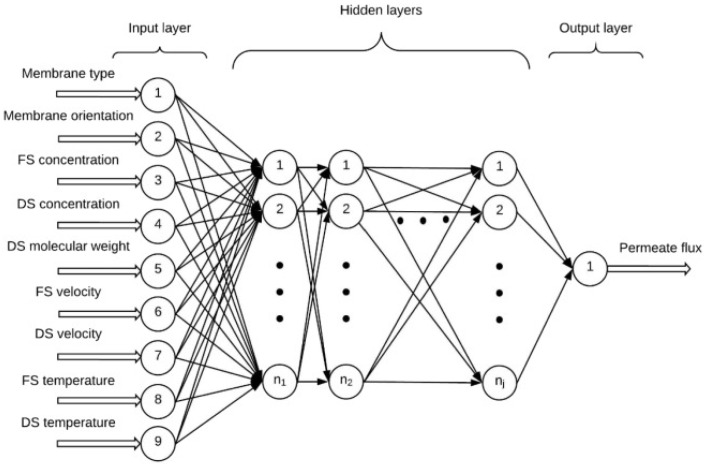
ANN network architecture with nine distinct input variables [136]. Reproduced with permission from Jawad J. et al., Desalination (2020), 484, 114427. Copyright © 2020 Elsevier License number: 6060270922137.

Mirbagheri et al. [133] conducted a study on the effect of simultaneous upward and downward aeration on membrane fouling and process performance in a submerged membrane bioreactor (SMBR). They used ANNs and genetic algorithms (GA) to simulate transmembrane pressure (TMP) and membrane permeability (Perm). The results indicated that simultaneous aeration did not significantly improve contaminant removal efficiency. The TMP increased and the Perm decreased with operational time. Comparing upward and simultaneous aeration, the TMP increasing rate and Perm decreasing rate were found to be 2.13 and 2.66 times higher for upward aeration, respectively. The GA-optimized ANN models accurately predicted TMP and permeability. Overall, it was observed that employing a membrane module with a simultaneous upward and downward aeration system resulted in significantly lower rates of TMP increase and permeability decrease compared to a single upward aeration system. The reduction in these rates could reach up to five times when utilizing the simultaneous aeration approach. S. Park et al. [134] presented a deep neural network (DNN) model for predicting membrane fouling during nano-filtration (NF) and reverse osmosis filtration using in situ fouling image data from optical coherence tomography (OCT). The DNN model was trained to simulate both organic fouling growth and flux decline, and it outperformed existing mathematical models in terms of predictive accuracy. The study also included image pre-processing to obtain accurate fouling information from OCT images and demonstrated good agreement between the model and observational data. The authors highlighted the potential of deep learning models in accurately predicting fouling behavior and improving the efficiency and sustainability of water treatment processes. S. Kamrava [135] employed a two-step approach to predict fluid flow and transport in complex porous membranes and materials using deep learning. First, they combined physics-based models with experimental image data to create a training dataset. The physics-based models provided the fundamental equations governing fluid flow and transport, while the experimental images captured the complex geometries and structures of the porous media. For the deep learning architecture, the authors utilized a combination of CNNs and recurrent neural networks (RNNs). The CNNs were used to extract spatial features from the image data, capturing the intricate details of the porous media. The RNNs, on the other hand, accounted for the temporal dependencies in the fluid flow and transport processes. The trained deep-learning model was then used to predict fluid flow and transport properties in unseen porous materials. The model demonstrated promising results, outperforming traditional physics-based models in terms of accuracy. It successfully predicted various properties such as permeability, porosity, and tortuosity, which are crucial for understanding fluid dynamics in porous materials. Jawad et al. [136] presented the application of artificial neural networks for the prediction of permeate flux in forward osmosis processes. The team constructed a multi-layered ANN model, incorporating nine distinct input variables: membrane type, membrane orientation, molarity, and type of both feed and draw solutions, crossflow velocity of feed and draw solutions, and the temperature of feed and draw solutions. Lab-scale experimental data from various previous studies confirmed the validity of the model. The ANN model exhibited a high degree of accuracy, with an R^2^ value of 97.3%, and demonstrated superior capability in forming complex relationships between inputs and outputs compared to multiple linear regression (MLR) models. As depicted in Figure 10, the performance was found to improve with a higher number of neurons per layer, while increasing the number of hidden layers beyond a certain point reduced accuracy. An optimal balance of more neurons and fewer hidden layers led to the highest predictive accuracy (R^2^ = 97.3%). The authors concluded that the ANN model, given its ability to predict lab-scale forward osmosis experiments within the data range used for training, offers a universal model for such predictions.

H. Gao et al. [137] delved into the utilization of machine-learning methodologies for addressing membrane fouling in UF systems. This team constructed ML algorithms to forecast membrane characteristics such as the average pore diameter, total porosity, and contact angle, with the fabrication parameters serving as the input variables. The robustness of these ML algorithms was corroborated by creating three distinct UF membranes and juxtaposing the empirical outcomes with the forecasted values, demonstrating a significant correlation. The constructed ML algorithms demonstrated their reliability in forecasting membrane performance based on chosen input variables. Nevertheless, the model’s predictive capacity was constrained for scenarios involving new additives that were not part of the training dataset.

So, ML is crucial in membrane fouling, providing novel methods for forecasting and comprehending membrane functionality. It facilitates precise predictions of membrane attributes and performance, thereby improving the design and efficiency of ultrafiltration membranes. Therefore, the integration of ML in this domain is not only beneficial but also essential for advancing membrane technology and mitigating fouling issues.

## 7. AI and ML in Membrane Characterization and Design

**Figure 11:**
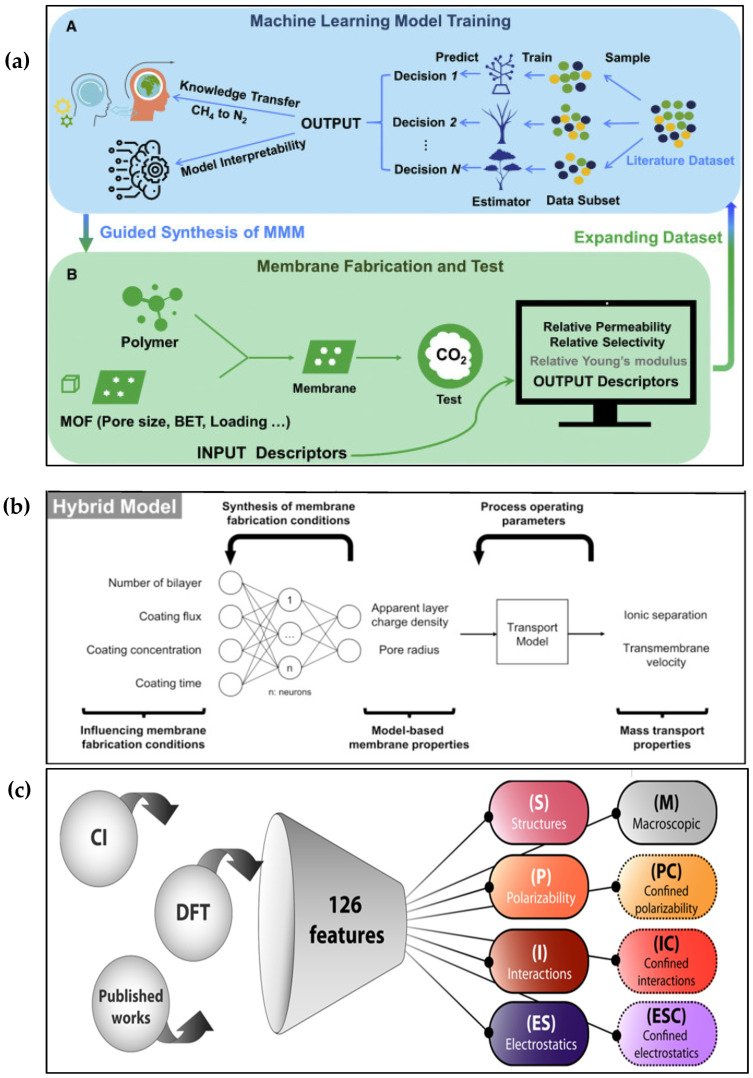
The proposed research paradigm for AI-accelerated membrane design and discovery, (**a**) Guan J. et al. [138] proposed an ML-guided framework for fabricating mixed matrix membranes (MMMs) (Reproduced with permission from Guan J. et al., Cell Reports Physical Science (2022), 3, 100,864 under the Creative Commons Attribution 4.0 License (CC BY 4.0), published by Cell Press. License available at: https://creativecommons.org/licenses/by/4.0/). (**b**) Deniz et al. [139] developed a hybrid model that combines different ML algorithms (Reproduced with permission from Rall D. et al., Journal of Membrane Science (2019), 569. 209–219., Copyright © 2019 Elsevier) License number: 6060330323027. (**c**) Features are collected and classified in ML-enabled knowledge extraction [140] (Reproduced with permission from Ritt C. et al., Sci. Adv. (2022), 8, eabl5771, under the terms of the Creative Commons Attribution Noncommercial License 4.0 (CC BY-NC), published by the American Association for the Advancement of Science (AAAS).

Membrane design and discovery involve complex interactions between membrane structure, properties, performance, and operating conditions, which are often difficult to predict and optimize by conventional methods. AI and ML can help overcome these challenges by analyzing large amounts of data, learning from existing knowledge, and generating novel insights and solutions. H. Yin et al. [30] presented the best practices for using AI methods for specific membrane design and discovery goals. They introduced a new research paradigm that uses AI to speed up membrane design and discovery, which has four steps: generating data, analyzing data, designing with data, and validating with experiments. They showed an example of this paradigm by designing a high-performance membrane for CO_2_ capture from flue gas. They also gave some general tips and advice for using AI methods in membrane research, such as picking suitable ML algorithms, selecting relevant features and descriptors, validating and interpreting the ML models, and ensuring data quality and availability. They emphasized the need for interdisciplinary collaboration between AI and membrane researchers to address the challenges and limitations of AI applications in membrane discovery. They also pointed out some future directions and opportunities for AI-assisted membrane innovation, such as incorporating domain knowledge and physical laws into ML models, developing hybrid ML models that combine different types of data and algorithms, exploring new membrane materials and structures using generative ML models, and applying AI methods to optimize membrane fabrication and operation processes. Figure 11 shows how different stages of the membrane discovery cycle, such as membrane material design, membrane fabrication, membrane operation, and knowledge extraction, can benefit from ML applications.

R. Maleki et al. [141] provided a comprehensive review of AI and its role in materials discovery and ion-selective membranes (ISMs) engineering. The author discussed the data pre-processing and feature engineering steps that are essential for AI-based materials discovery. The paper also reviewed the different types of AI methods, such as supervised learning, unsupervised learning, reinforcement learning, and deep learning, and their advantages and disadvantages for materials discovery, especially in membrane technology and design. The authors also presented a hybrid method that combines AI with computational chemistry to consider atomic features in the output models and then focused on the applications of AI in ISMs engineering, such as membrane material design, membrane fabrication, membrane operation, and knowledge extraction. R. Salehi et al. [142] studied the application of ML models to predict the performance of a cross-flow ultrafiltration membrane in xylose reductase separation. The authors employed two advanced ML models, namely the Adaptive Neuro-Fuzzy Inference System based on Grid Partitioning (ANFIS-GP) and Boosted Regression Trees (BRT), and compared their predictive performances with that of the best nonlinear multiple regression picked from among 143 different regressions, and of linear multiple regression. The study found that the BRT model outperformed the ANFIS-GP model, offering the highest R^2^ and NSE values and producing the least error. However, the authors noted that BRT models are prone to instability and overfitting and are computationally expensive. The sensitivity analysis for the BRT model showed that the input variable X1 (filtration time) has the highest impact on the model output, followed by input variables X2 (transmembrane pressure) and X3 (cross-flow velocity), showing a lower impact. The paper concluded that while the developed models demonstrated meaningful predictive power, there is a need for model calibration and validation. D. H. Kang et al. [143] developed a geometric feature map-based CNN to predict the average surface roughness of nanofiber membranes directly from SEM images. The model integrated three feature maps—contrast-limited adaptive histogram equalization (CLAHE), binarization, and two-dimensional discrete Fourier transform (2D-DFT)—to extract geometric patterns describing fiber distribution and pore characteristics. Using a dataset of 71 SEM images paired with atomic force microscopy (AFM) roughness values, the CNN achieved high predictive accuracy (R^2^ = 0.979; MAPE = 4.8%) across a wide roughness range from 3 nm to 3 µm. Similarly, J. Tang et al. [144] introduced a hybrid ML model that integrates genetic algorithm–optimized backpropagation neural networks (GA-BPNN) with fractal geometry to predict cake layer permeability on carbon nanotube (CNT) nanocomposite membranes. Using 3D confocal laser scanning microscopy (CLSM) and pore network modeling, structural parameters such as pore space fractal dimension, anisotropy, and porosity were extracted as key predictors. The GA-BPNN achieved exceptional predictive performance (R^2^ = 0.99 for training and 0.96 for testing), surpassing conventional BPNN and classical Kozeny–Carman models. The study revealed that membrane surface fractality (Df = 2.11–2.59) significantly affects cake morphology and fouling behavior, with intermediate fractal dimensions promoting higher permeability and better pore connectivity. X. Deng et al. [145] proposed an integrated phase-field modeling and ANN framework to predict and optimize the microstructure and electrochemical performance of polymer membranes for alkaline water electrolysis. By coupling phase-field simulations with fully connected and convolutional neural networks, the model accurately predicted key structural parameters—tortuosity and maximum pore size—with R^2^ values of 0.75 and 0.89, respectively. Sensitivity analyses revealed that polymer concentration and solvent–nonsolvent affinity strongly influence membrane morphology and transport properties. The optimized membranes exhibited enhanced ion conductivity and gas-barrier performance, underscoring the potential of ML-assisted microstructure modeling for rational membrane design. In another study, S. J. Im et al. [146] trained separated ANN models, to predict fouling characteristics (thickness, porosity, roughness, density), water flux, and pollutant removal (DOC, TN, TP) using the same water quality inputs. Nine input variables, such as dissolved organic carbon (DOC), UV_(254)_, proteins, polysaccharides, and ion concentrations (Na^+^, Ca^2+^, Cl^−^), were analyzed to identify optimal predictors. The ANN model architecture (2–4 hidden layers, 10–15 neurons) achieved high accuracy, with R^2^ values of 0.85–0.98 for fouling properties, 0.92 for flux prediction, and 0.87–0.92 for organic and nutrient removal. These results demonstrate that ANN can effectively capture nonlinear relationships in FO systems, offering a powerful tool for predictive control and fouling management in wastewater reuse applications (Table 2).

Overall, the reviewed studies collectively illustrate how the integration of AI and ML has transformed membrane characterization and design from empirical trial-and-error approaches into predictive, data-driven engineering. Early works primarily focused on structure–property correlations using regression and tree-based models, while recent advances have embraced deep and hybrid neural network frameworks capable of extracting complex spatial and physicochemical features from multi-modal datasets such as SEM, CLSM, and phase-field simulations. This evolution demonstrates a clear shift toward hybrid modeling—where experimental, computational, and image-derived data are synergistically combined to capture nonlinear relationships between membrane morphology, transport properties, and fouling dynamics. Models such as CNNs, GA-BPNNs, and hybrid ANNs have proven particularly effective in predicting surface roughness, fractal-based permeability, and microstructural tortuosity with high accuracy (R^2^ up to 0.99). Furthermore, studies integrating ML with fractal geometry and phase-field modeling highlight the growing capacity of AI to rationalize hierarchical membrane structures and optimize electrochemical and filtration performance. Collectively, these advancements position AI and ML as indispensable tools for the next generation of membrane materials—enabling intelligent, automated design strategies that bridge nanoscale structure with macroscopic functionality.

## 8. Challenges and Future Directions: AI and ML in Membrane Science

AI and ML have demonstrated substantial potential in advancing membrane engineering, particularly in areas such as novel material discovery, membrane fabrication process optimization, fouling behavior prediction, and performance modeling. Despite these advancements, several domain-specific challenges must be addressed to fully harness their capabilities in membrane technologies. Below are key challenges and prospective research directions tailored to this field:

1. Data Quality and Availability: AI and ML algorithms are heavily reliant on large, high-quality datasets for training and validation. In membrane engineering, experimental data on permeability, selectivity, fouling resistance, and material composition are often scarce or inconsistently reported. The labor-intensive nature of membrane characterization (e.g., SEM, FTIR, AFM, zeta potential measurements) further limits dataset generation. Hence, the development of standardized data formats, membrane-specific open-access repositories, and strategies for data augmentation and fusion (e.g., combining simulation and experimental data) is urgently needed [147].
2. Model Validation and Interpretation: While ML models can effectively capture the nonlinear behavior of membrane performance (e.g., flux versus pressure, solute rejection versus pore size), their “black box” nature often hinders adoption in engineering design. In membrane development, interpretability is essential to understand structure–property relationships. As such, rigorous validation using membrane module testing, cross-lab reproducibility, and explainable AI (XAI) approaches are critical to ensure trustworthiness and facilitate regulatory or industrial adoption [148].
3. Integration of Domain Knowledge: While AI and ML methods can learn from data without prior knowledge or assumptions, they can also benefit from incorporating domain knowledge and physical laws into their models. Membrane systems are governed by complex physical and chemical phenomena such as Donnan exclusion, concentration polarization, and hydrodynamic shear stress. Hybrid ML models that incorporate transport equations, sorption models, or empirical correlations can improve generalization, especially under extrapolative conditions. Embedding such physics-informed priors or constraints can also reduce the need for large datasets and prevent overfitting in membrane-specific applications.
4. Generative Models: AI and ML methods are commonly used for predictive and descriptive modeling to infer outputs from inputs or identify patterns in data. Recently, generative approaches—such as Generative Adversarial Networks (GANs) and Variational Autoencoders (VAEs)—have emerged as powerful tools for inverse design, enabling the creation of novel membrane materials or surface modifications that have not yet been experimentally tested. These models can accelerate the discovery of membranes with tailored physicochemical properties, such as enhanced antifouling resistance, improved selectivity, or tunable hydrophilicity, by efficiently exploring the material design space beyond the limitations of conventional trial-and-error experimentation [149].
5. Optimization Methods: AI and ML techniques can be used to solve complex optimization problems by identifying optimal solutions under specific objectives and constraints. In membrane engineering, these methods can assist in optimizing fabrication parameters—such as solvent ratios, casting speed, and annealing temperature—as well as operational conditions, including pressure cycles, cleaning intervals, and feed temperature. Additionally, optimization algorithms (e.g., Bayesian optimization, evolutionary algorithms) can be employed to tune hyperparameters or architectures of predictive models, thereby improving generalization and model performance. These approaches are particularly valuable for addressing nonlinear, multi-objective optimization challenges commonly encountered in membrane material design and process development. In addition to these directions, promising strategies include Hybrid modeling that combines AI/ML with classical transport models (e.g., solution-diffusion or pore flow) to achieve both predictive accuracy and interpretability/Active learning frameworks that can reduce the experimental burden by selecting the most informative data points—especially useful in fouling studies or degradation scenarios where data collection is time-intensive.

In conclusion, the integration of AI and ML into membrane engineering presents numerous opportunities to advance the field. Addressing the challenges of data quality, interpretability, and domain integration will be essential to unlock the full potential of these technologies. As methods mature, they are expected to drive innovation in membrane material discovery, performance prediction, and system optimization, leading to more efficient and intelligent membrane-based processes.

## 9. Conclusions

The integration of AI and ML into material science and membrane technology represents a transformative shift from traditional empirical approaches to data-driven innovation. This review has highlighted how these technologies have advanced every stage of the membrane lifecycle—from material discovery and fabrication to process modeling, fouling control, and performance optimization. By accelerating development cycles, reducing experimental costs, and revealing hidden structure–property relationships, AI and ML have become indispensable tools for next-generation membrane design. Despite this progress, challenges persist, particularly regarding data availability, model interpretability, and the incorporation of domain-specific physical knowledge. Addressing these challenges through standardized datasets, hybrid modeling frameworks, and interpretable AI systems will be crucial for maximizing the potential of AI/ML in this field. Moving forward, close collaboration among material scientists, chemical engineers, and data scientists will be essential to build transparent, physics-informed, and scalable solutions.

In summary, the novelty of this review lies in its holistic integration of AI and ML across the entire membrane research spectrum. Unlike previous reviews limited to specific topics such as process optimization or fouling control, this work establishes a cross-disciplinary synthesis linking material informatics and membrane engineering. It critically evaluates current advances, identifies limitations, and proposes a unified data-driven framework that can guide future AI-assisted membrane research and innovation.

## Figures and Tables

**Table 2:** Overview of supervised ML models used in membrane design and analysis.

ML Model	Learning Type	Membrane Application	Input Features	Output/ Target Variable	Performance Metrics	Key Finding	Remarks/ Challenges	Ref.
CNN	Supervised (Deep Learning—Regression)	Prediction of average surface roughness of nanofiber membranes	Preprocessed SEM images (grayscale, CLAHE, binarization, 2D Discrete Fourier Transform) capturing. geometric features such as fiber diameter, pore fraction, and pixel intensity gradients	Logarithmic average surface roughness (log Ra)	Mean Absolute Percentage Error (MAPE) = 4.8%; R^2^ = 0.979 (test)	CNN model accurately predicted surface roughness across a 3 nm–3 µm range, geometric feature extraction	Out-of-range prediction challenges for smooth surfaces (<30 nm); trade-off between accuracy and inference time	[143]
GA-BPNN	Supervised (Regression)	Prediction of K_f_ on CNT nanocomposite membranes	Pore space fractal dimension (2.44–2.81), pore anisotropy (0.64–0.81), porosity (0.30–0.71)—extracted from 3D CLSM imaging and pore network modeling	K_f_	R^2^ = 0.99 (training), R^2^ = 0.96 (testing); RMSE ≈ 0.13	Demonstrated strong correlation between membrane surface fractality and fouling behavior; GA-BPNN hybrid modeling accurately predicts cake permeability and outperforms classical models	Limited dataset and material scope; generalization to other membranes and dynamic fouling conditions requires further validation.	[144]
Fully Connected Neural Network (FCNN) and CNN	Supervised (Regression)	Prediction and optimization of membrane pore structure for alkaline water electrolysis	Polymer concentration (wt%), solvent/ nonsolvent ratio, interaction parameters (χsp, χnp, χns), diffusion coefficients, and coagulation bath composition	Tortuosity and maximum pore size	R^2^ = 0.75 (tortuosity), R^2^ = 0.89 (pore size)	Coupling phase-field simulation with ML enables accurate prediction of microstructure parameters; polymer concentration and solvent–nonsolvent affinity strongly affect tortuosity and pore size.	Limited dataset; extension to other polymers and electrochemical systems required for broader generalization.	[145]
ANN (Feed-forward, trained with Levenberg–Marquardt algorithm)	Supervised (Regression)	Prediction of FO membrane fouling, water flux, and pollutant removal performance	DOC, UV_254_, TN, TP, Ca^2+^, Na^+^, Cl^−^, proteins, polysaccharides (real wastewater data)	Fouling properties: thickness, porosity, roughness, density Flux: water flux Removal: DOC, TN, TP removal	R^2^ = 0.85–0.98 (fouling) R^2^ = 0.92; RMSE = 0.9 L·m^−2^·h^−1^ (flux) R^2^ = 0.87–0.92; error ≤ 2.7% (removal)	ANN accurately predicted fouling behavior, flux, and removal efficiencies; highest precision for fouling properties (R^2^ ≈ 0.98).	Limited dataset (single FO configuration); model generalization to other feeds and scales requires validation	[146]

## Data Availability

No new data were generated in this review article.
